# Safe Use and Storage of Cleaners, Disinfectants, and Hand Sanitizers: Knowledge, Attitudes, and Practices among U.S. Adults during the COVID-19 Pandemic, May 2020

**DOI:** 10.4269/ajtmh.20-1119

**Published:** 2020-12-29

**Authors:** Radhika Gharpure, Gabrielle F. Miller, Candis M. Hunter, Amy H. Schnall, Jasen Kunz, Amanda G. Garcia-Williams

**Affiliations:** 1 COVID-19 Response, U.S. Centers for Disease Control and Prevention, Atlanta, Georgia;; 2 National Center for Environmental Health, U.S. Centers for Disease Control and Prevention, Atlanta, Georgia

## Abstract

Cleaning and disinfection of frequently touched surfaces and frequent hand hygiene are recommended measures to prevent transmission of SARS-CoV-2, the virus that causes COVID-19. Since the onset of the COVID-19 pandemic, poison center calls regarding exposures to cleaners, disinfectants, and hand sanitizers have increased as compared with prior years, indicating a need to evaluate household safety precautions. An opt-in Internet panel survey of 502 U.S. adults was conducted in May 2020. Survey items evaluated knowledge regarding use and storage of cleaners, disinfectants, and hand sanitizers; attitudes about household cleaning and disinfection; and safety precautions practiced during the prior month. We assigned a knowledge score to each respondent to quantify knowledge of safety precautions and calculated median scores by demographic characteristics and attitudes. We identified gaps in knowledge regarding safe use and storage of cleaners, disinfectants, and hand sanitizers; the overall median knowledge score was 5.17 (95% CI: 4.85–5.50; maximum 9.00). Knowledge scores were lower among younger than older age-groups and among black non-Hispanic and Hispanic respondents compared with white non-Hispanic respondents. A greater proportion of respondents expressed knowledge of safety precautions than the proportion who engaged in these precautions. Tailored communication strategies should be used to reach populations with lower knowledge of cleaning and disinfection safety. In addition, as knowledge alone did not shape individual engagement in safety precautions, health promotion campaigns may specifically emphasize the health risks of unsafe use and storage of cleaners, disinfectants, and hand sanitizers to address risk perception.

## INTRODUCTION

Cleaning and disinfection of frequently touched surfaces are recommended measures to prevent transmission of SARS-CoV-2, the virus that causes COVID-19.^1^ Although the virus is thought to primarily spread through respiratory droplets, it can survive on surfaces from hours to days,^2^ allowing for potential fomite transmission from contact with frequently touched surfaces and subsequent contact with mucous membranes. Frequent hand hygiene, consisting of handwashing with soap and water for at least 20 seconds or using a hand sanitizer containing at least 60% alcohol, is also a key prevention measure to interrupt SARS-CoV-2 transmission^1^ in addition to measures such as physical distancing and mask wearing.

Several studies conducted in the United States, United Kingdom, and Australia have indicated that a large proportion of adults have engaged in more frequent household cleaning and disinfection and hand hygiene since the start of the COVID-19 pandemic.^3–7^ A survey of U.S. adults conducted in March 2020 found that most respondents agreed that disinfecting surfaces, handwashing, and hand sanitizing could slow the spread of COVID-19.^5^ However, to ensure that cleaning and disinfection activities and use of hand sanitizer products do not result in unintended chemical exposures and health hazards,^8^ safety precautions such as following label directions, appropriate preparation of cleaning and disinfectant solutions, use of recommended skin and eye protection when using cleaners and disinfectants, and safe storage of cleaning, disinfection, and hand sanitizer products away from children and pets are critical.^9^ Data from U.S. poison centers have shown a rise in calls related to exposures to cleaners, disinfectants, and hand sanitizers since the onset of the COVID-19 pandemic as compared with prior years,^10^ and similar rises in poison center calls have been described in France,^11^ Italy,^12^ and Croatia.^13^

Studies evaluating household cleaning and disinfection safety are limited, particularly during the COVID-19 pandemic. Before the pandemic, a study assessing consumer exposures to household chemicals in the United States found that safety precautions such as use of gloves were more commonly reported by female than male participants and that only a very small proportion (5%) of participants read product safety labels.^14^ In a study assessing women’s household cleaning practices in Lebanon, similar gaps were identified in reading label instructions and use of gloves, as well as in appropriate mixing of chemicals; participants expressed that the importance of cleanliness and hygiene was a stronger priority than concern for exposure to chemicals.^15^ Prior studies conducted in Switzerland found that consumers’ risk perceptions of different cleaning and disinfectant products were shaped by the products’ label instructions, intended use, perceived effectiveness, and marketing of qualities such as “eco-friendliness,”^16^ and that risk perceptions predicted engagement in safety behaviors.^17^ Another Swiss study found that participants overestimated their own adherence to safety practices, whereas 85% of participants considered their behavior to be safer than the behavior of the average consumer, 71% stored cleaning or disinfection products at a height accessible to children, and 36% did not store products in a closed cabinet.^18^

Given the need to better characterize public engagement in safety precautions for use and storage of household cleaners, disinfectants, and hand sanitizers during the COVID-19 pandemic, a rapid turnaround survey of U.S. adults was conducted.^19^ Initial findings included increased frequency of household cleaning and disinfection by a majority (60%) of respondents; gaps in knowledge about safe preparation, use, and storage of products; and use of cleaners and disinfectants in non-recommended, high-risk practices.^19^ To further examine these survey data, the objectives of this study were to 1) characterize demographic predictors of knowledge regarding safe use and storage of cleaners, disinfectants, and hand sanitizers; 2) understand how attitudes were associated with knowledge; and 3) characterize gaps between knowledge and practices.

## MATERIALS AND METHODS

### Survey instrument and data collection.

Survey data were collected by Porter Novelli (PN) Public Services and ENGINE Insights on May 5–7, 2020 via “PN View: 360,” an opt-in Internet panel of 502 U.S. adults aged 18 years and older.^19^ The survey was conducted using the Lucid platform^20^; quota sampling was used, and panel members who had not taken a survey in the previous 20 waves of survey administration were eligible to participate. Four survey items were included in this analysis (Supplemental Materials). The first survey item asked about knowledge of cleaning and disinfection safety; responses regarding storage of hand sanitizers were included along with responses regarding storage of cleaners and disinfectants. This item was measured using a binary scale (checked or unchecked). The second survey item asked about cleaning and disinfection safety precautions practiced during the prior month; this was also measured using a binary scale. The third and fourth survey items evaluated attitudes (including self-efficacy, norms, and outcome expectancies) about cleaning and disinfection safety; these were measured using a five-point Likert-type scale ranging from one (strongly disagree) to five (strongly agree). Seven additional survey items regarding behaviors specifically intended to prevent SARS-CoV-2 transmission and self-reported health outcomes were administered but were not included in this analysis. For all survey items, the term “cleaning” was used to encompass both cleaning and disinfection in plain language. Data quality filters designed to prevent cheating or speeding were embedded in the survey. Respondents were informed that their answers were being used for market research and could refuse to answer any question at any time.

Data were weighted by gender, age, race/ethnicity, region, and education to reflect the composition of the U.S. population using the Current Population Survey proportions from the U.S. Census Bureau and U.S. Bureau of Labor Statistics. The CDC obtained the survey data from Porter Novelli Public Services through a subscription license, and no personally identifiable information was included in the provided data file. Porter Novelli Public Services and its vendors are not subject to review by CDC’s Institutional Review Board. This activity was deemed not to be research as defined in 45 Code of Federal Regulations 46.102(l), and a Paperwork Reduction Act waiver was obtained as the data collection was part of the CDC COVID-19 emergency response.

### Knowledge score construction.

We assigned a knowledge score to each respondent to characterize the overall knowledge of cleaning and disinfection safety precautions based on the following survey item: “Which of the following have you heard is true about using household cleaning products? (select all that apply).” The knowledge score was calculated by assigning a value of “one” to each checked response and “zero” to each unchecked response. These values were summed across nine possible response options; thus, the highest possible knowledge score was nine, and the lowest score was zero. The following nine response options were included: 1) “For some household cleaning products, gloves should be used during use”; 2) “For some household cleaning products, eye protection should be used during use”; 3) “Bleach should not be mixed with ammonia”; 4) “Bleach should not be mixed with vinegar”; 5) “When making a dilute bleach solution, only room temperature water should be used”; 6) “Hands should be washed with soap and water after using household cleaning products”; 7) “Hand sanitizers should be kept out of reach of children”; 8) “Household cleaning products should be kept out of reach of children”; and 9) “Good ventilation (air flow) is needed when using cleaning chemicals.”

### Data analysis.

We calculated median knowledge scores and 95% CIs by demographic characteristics, including gender (male and female), age-group (18–24 years, 25–34 years, 35–44 years, 45–54 years, 55–64 years, and 65+ years), race/ethnicity (white non-Hispanic, black non-Hispanic, other non-Hispanic, and Hispanic [of any race]), U.S. Census geographic region (Northwest, Midwest, South, and West), education (high school or less, high school graduate, some college, and college graduate), and urbanicity (urban, suburban, and rural). Likert-type attitudinal response items were dichotomized to assess agreement (agreed = strongly agree and somewhat agree; disagreed or neutral = strongly disagree, somewhat disagree, and neutral) and median knowledge scores, and 95% CIs were calculated for these responses. All analyses were conducted using SAS statistical software (version 9.4; SAS Institute Inc., Cary, NC), accounting for the complex sampling design and sampling weights. Unweighted sample sizes (*n*) and weighted percentages (%) are reported. CI overlap was used to determine if results were significant; no additional inferential statistics (e.g., *P*-values) were calculated because of limitations of non-probability sampling.^21^

## RESULTS

### Respondent demographics.

Survey respondents included 502 U.S. adults with age ranging from 18 to 86 years; the median age of respondents was 46 years, and approximately half (52%) were female (Table 1). Of all respondents, 64% were white non-Hispanic, 12% were black non-Hispanic, 8% were other non-Hispanic, and 16% were Hispanic of any race. In addition, 18% of respondents were from the Northeast U.S. Census geographic region, 21% from the Midwest, 38% from the South, and 24% from the West. Education levels of respondents included high school or less (5%), high school graduates (35%), some college education (27%), and college graduates (34%). Finally, approximately half of respondents (49%) were from suburban communities, whereas 31% were from urban communities and 20% were from rural communities.

**Table 1:** Demographic characteristics of survey respondents* (*n* = 502) and knowledge of cleaning and disinfection safety precautions, United States, May 2020

Characteristic	*N* (weighted %†)	Knowledge score,‡ median (95% CI)
Total	502 (100)	5.17 (4.85–5.50)
Gender		
Male	251 (48)	4.71 (4.16–5.27)
Female	251 (52)	5.50 (5.06–5.94)
Age-group (years)		
18–24	56 (12)	3.80 (2.57–5.02)
25–34	102 (18)	3.36 (2.59–4.12)
35–44	106 (16)	4.63 (3.62–5.63)
45–54	66 (17)	5.48 (4.80–6.17)
55–64	88 (17)	6.10 (5.52–6.68)
65+	84 (21)	5.85 (5.11–6.59)
Race/ethnicity		
White non-Hispanic	342 (64)	5.59 (5.24–5.94)
Black non-Hispanic	51 (12)	2.99 (1.61–4.37)
Other non-Hispanic	50 (8)	4.42 (3.29–5.54)
Hispanic (any race)	59 (16)	4.01 (2.80–5.21)
U.S. Census geographic region		
Northeast	93 (18)	5.23 (4.51–5.95)
Midwest	102 (21)	5.38 (4.77–5.98)
South	187 (38)	5.10 (4.55–5.65)
West	120 (24)	5.03 (4.11–5.95)
Education		
High school or less	18 (5)	4.31 (1.89–6.72)
High school graduate	142 (35)	5.31 (4.60–6.01)
Some college	132 (27)	5.36 (4.69–6.03)
College graduate	210 (34)	4.97 (4.35–5.59)
Urbanicity		
Urban	154 (31)	4.28 (3.54–5.01)
Suburban	253 (49)	5.35 (4.91–5.79)
Rural	95 (20)	5.79 (4.78–6.80)

* Respondents were surveyed through the “PN View: 360” opt-in Internet panel survey administered from May 5 to 7, 2020.

† Percentages may not sum to 100 because of rounding. Data were weighted by gender, age, race/ethnicity, region, and education to reflect the composition of the U.S. population using the Current Population Survey proportions.

‡ The highest possible score was nine. The knowledge score was calculated by assigning a score of one to each of the following checked responses and zero to each unchecked response: 1) “For some household cleaning products, gloves should be used during use”; 2) “For some household cleaning products, eye protection should be used during use”; 3) “Bleach should not be mixed with ammonia”; 4) “Bleach should not be mixed with vinegar”; 5) “When making a dilute bleach solution, only room temperature water should be used”; 6) “Hands should be washed with soap and water after using household cleaning products”; 7) “Hand sanitizers should be kept out of reach of children”; 8) “Household cleaning products should be kept out of reach of children”; and 9) “Good ventilation (air flow) is needed when using cleaning chemicals.”

### Reported knowledge of safe cleaning, disinfection, and hand sanitizer storage.

The median knowledge score was 5.17 (95% CI: 4.85–5.50; maximum 9.00) across all respondents (Table 1). Knowledge of the specific nine safety precautions that comprised the knowledge score were previously reported^19^; the proportion of respondents who correctly selected each response ranged from 23% (“When making a dilute bleach solution, only room temperature water should be used”) to 79% (“Household cleaning products should be kept out of reach of children”). Knowledge scores varied by demographic characteristics (Table 1). Specifically, knowledge scores were lower among younger age-groups than among older age-groups; respondents aged 18–24 years (median 3.80 [95% CI: 2.57–5.02]) had lower scores than those aged 55–64 years (median 6.10 [95% CI: 5.52–6.68]) and 65+ years (median 5.85 [95% CI: 5.11–6.59]), and respondents aged 25–34 years (3.36 [95% CI: 2.59–4.12]) had lower scores than those aged 45–54 years (median 5.48 [95% CI: 4.80–6.17]), 55–64 years, and 65+ years. Furthermore, knowledge scores were lower among black non-Hispanic respondents (median 2.99 [95% CI: 1.61–4.37]) and Hispanic respondents (median 4.01 [95% CI: 2.80–5.21]) than among white non-Hispanic respondents (median 5.59 [95% CI: 5.24–5.94]).

### Knowledge and attitudes.

Attitudes about safe cleaning and disinfection correlated with knowledge scores (Table 2). Knowledge scores were higher among respondents who agreed that they knew how to clean and disinfect their homes safely (median 5.44 [95% CI: 5.09–5.78]), were able to clean and disinfect safely (median 5.44 [95% CI: 5.09–5.78]), and were able to store cleaning products safely (median 5.44 [95% CI: 5.09–5.79]) than among those who did not agree with these statements. Similarly, respondents who agreed that they knew where to get information on safe cleaning behaviors had higher knowledge scores (median 5.39 [95% CI: 5.04–5.74]) than those who did not agree (median 3.86 [95% CI: 2.93–4.80]). Respondents who agreed that misuse of household cleaners and disinfectants can result in injury had higher scores (median 5.41 [95% CI: 5.09–5.73]) than those who did not agree (median 2.03 [95% CI: 0.93–3.10]).

**Table 2:** Attitudes and knowledge of cleaning and disinfection safety precautions among survey respondents* (*n* = 502), United States, May 2020

Statement†	Agreed		Disagreed or neutral	
	*N* (weighted %‡)	Knowledge score§	*N* (weighted %‡)	Knowledge score§
		median (95% CI)		median (95% CI)
I know how to clean and disinfect my home safely.	408 (81)	5.44 (5.09–5.78)	94 (19)	3.21 (2.22–4.20)
I am able to clean and disinfect my home safely.	413 (82)	5.44 (5.09–5.78)	89 (18)	3.35 (2.24–4.46)
I know how to store cleaning products in my home safely.	422 (84)	5.44 (5.09–5.79)	80 (16)	2.85 (1.81–3.90)
I know where to get information on safe cleaning behaviors.	388 (77)	5.39 (5.04–5.74)	114 (23)	3.86 (2.93–4.80)
I am able to buy or locate the household cleaning products I need to clean my home.	380 (75)	5.33 (4.99–5.67)	122 (25)	4.22 (3.14–5.30)
People I care about want me to clean my home safely.	372 (73)	5.37 (4.99–5.75)	130 (27)	4.42 (3.49–5.34)
Misuse of household cleaning products can result in injury.	434 (86)	5.41 (5.09–5.73)	68 (14)	2.03 (0.93–3.10)

* Respondents were surveyed through the “PN View: 360” opt-in Internet panel survey administered from May 5 to 7, 2020.

† Survey items were administered on a five-point Likert scale ranging from one (strongly disagree) to five (strongly agree) and dichotomized to assess agreement (agreed = strongly agree and somewhat agree; disagreed or neutral = strongly disagree, somewhat disagree, and neutral).

‡ Percentages may not sum to 100 due to rounding. Data were weighted by gender, age, race/ethnicity, region, and education to reflect the composition of the U.S. population using the Current Population Survey proportions.

§ The highest possible score was nine. The knowledge score was calculated by assigning a score of one to each of the following checked responses and zero to each unchecked response: 1) “For some household cleaning products, gloves should be used during use”; 2) “For some household cleaning products, eye protection should be used during use”; 3) “Bleach should not be mixed with ammonia”; 4) “Bleach should not be mixed with vinegar”; 5) “When making a dilute bleach solution, only room temperature water should be used”; 6) “Hands should be washed with soap and water after using household cleaning products”; 7) “Hand sanitizers should be kept out of reach of children”; 8) “Household cleaning products should be kept out of reach of children”; and 9) “Good ventilation (air flow) is needed when using cleaning chemicals.”

### Knowledge and practices.

**Figure 1.**
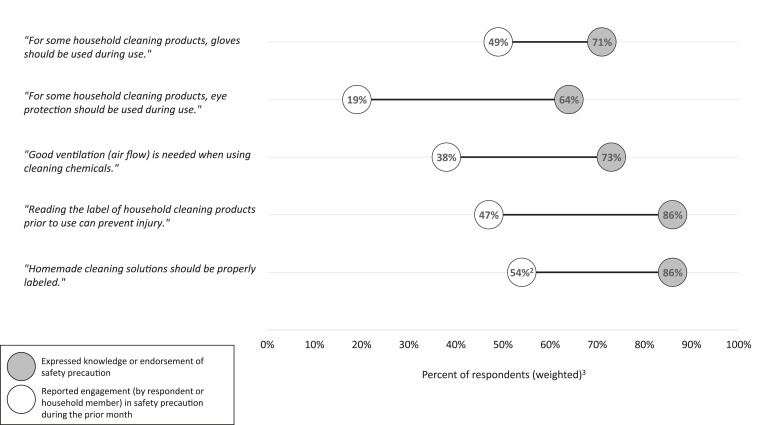
Gaps between knowledge and practice of cleaning and disinfection safety precautions among survey respondents^1^ (*n* = 502), United States, May 2020.^1^Respondents were surveyed through the “PN View: 360” opt-in Internet panel survey administered from May 5 to 7, 2020. ^2^Labeling of solutions was evaluated among 88 respondents who reported preparing a homemade solution in the prior month. ^3^Data were weighted by gender, age, race/ethnicity, region, and education to reflect the composition of the U.S. population using the Current Population Survey proportions.

A greater proportion of respondents expressed knowledge regarding use of gloves and eye protection, adequate ventilation, reading labels, and labeling homemade cleaning or disinfectant solutions than the proportion who reported that they or a household member had engaged in these safety precautions during the prior month (Figure 1). Specifically, although 71% (95% CI: 67–75) of respondents reported knowledge of recommendations regarding glove use for some cleaning and disinfection products, only 49% (95% CI: 44–53) reported using gloves during household cleaning and disinfection in the prior month. Similar gaps were apparent in the use of eye protection (64% [95% CI: 59–68] reported knowledge versus 19% [95% CI: 15–23] reported engagement), ventilation (73% [95% CI: 69–77] versus 38% [95% CI: 34–43]), and reading product labels (86% [95% CI: 82–89] versus 47% [95% CI: 42–51]). Finally, although 86% (95% CI: 82–89) of respondents endorsed recommendations regarding labeling of homemade solutions, only 54% (95% CI: 43–65) of respondents who reported preparing a homemade solution in the prior month reported labeling solutions.

## DISCUSSION

This survey found relatively limited knowledge of safety precautions for cleaners, disinfectants, and hand sanitizers. Knowledge scores varied by age and race/ethnicity, with lower knowledge scores among younger age-groups compared with older age-groups and among black non-Hispanic and Hispanic respondents compared with white non-Hispanic respondents. These findings align closely with previously reported disparities in other COVID-19–related knowledge. A recent national survey of U.S. adults conducted in March–April 2020 indicated that knowledge regarding the potential spread of COVID-19 via fomite transmission was lower among black and Hispanic respondents than among white respondents and among those younger than 55 years than among those who were older^22^; the study also identified similar disparities in knowledge regarding other modes of transmission and regarding COVID-19 symptoms. Collectively, these results indicate that these populations may not be reached effectively by current public messaging efforts regarding COVID-19 transmission, illness, and prevention, including information regarding how to engage in prevention measures safely. Racial and ethnic minority groups may be at increased risk of COVID-19 illness because of long-standing systemic inequities,^23^ and thus, addressing barriers to information regarding COVID-19 prevention is a public health priority.

Tailoring of prevention messages may increase their relevance to intended audiences, thereby increasing their effectiveness in behavior change interventions.^24^ Health communication strategies and channels tailored to varying languages, literacy levels, and cultures should be explored to reach groups with lower knowledge regarding COVID-19 prevention. In addition, efforts to promote health equity may include partnerships with community organizations, community members, and other key stakeholders to amplify messaging regarding safe and effective COVID-19 prevention. These efforts may include outreach, research, and development of strategies and policies to identify underlying needs and barriers related to messaging and COVID-19 prevention practices. For example, studies related to the social determinants of health have been prioritized to better contextualize disproportionate impacts of COVID-19.^25^

Respondents’ attitudes about safe cleaning and disinfection were associated with knowledge scores. This suggests that those with lower knowledge scores may not have been reached by prevention messaging; prevention messages can impact attitudes such as self-perceived knowledge and perceived ability to clean and disinfect safely, store products safely, and obtain information on safety precautions. This finding underscores the importance of making this information more readily and easily accessible to improve public knowledge regarding cleaning and disinfection safety precautions.

It is important to note that the proportions of respondents who reported engaging in safety precautions during the prior month were substantially lower than the proportions of those who expressed knowledge of these precautions. These findings indicate that knowledge alone did not shape individual engagement in practices. Further formative research is warranted to characterize barriers to engagement in safety precautions. Previous qualitative studies have indicated that consumers may not engage in safety precautions that are perceived to require a high burden of effort^18^; for example, in one study, parents of young children expressed that storing household chemicals out of reach of children was often not as convenient as storing them where easily accessible.^26^ However, having heard about or having encountered a child poisoning event was a significant motivator of adopting safety practices. Low levels of engagement in prevention measures (e.g., glove use and reading labels) were also observed among parents who were interviewed regarding household pesticide use^27^; participants expressed that when they perceived themselves to be more familiar with products, they were less likely to read and follow label instructions.

Understanding determinants that shape adherence to safety precautions for cleaning, disinfection, and hand sanitizer storage, particularly in the context of the COVID-19 pandemic, may facilitate development of effective health communication messages. Recent studies have indicated that risk awareness (e.g., having a close friend or relative who tested positive for SARS-CoV-2) was found to motivate more frequent effort in cleaning and disinfecting surfaces^6^ and engagement in hand hygiene.^28^ Similarly, increasing risk awareness specifically regarding unsafe cleaning and disinfection practices may increase adoption of safety precautions, as low risk perception may motivate lack of adherence to safety precautions. Prevention messages that describe examples of adverse health outcomes experienced by individuals who engaged in unsafe use or storage of cleaners, disinfectants, and hand sanitizers may be effective and should be further evaluated. Finally, supply disruptions for cleaners, disinfectants, hand sanitizers, and skin and eye protection should be explored as potential barriers to engaging in safety precautions during the COVID-19 pandemic.

These findings are subject to several limitations. First, this survey was based on an opt-in Internet panel sample, which can limit generalizability of knowledge, attitudes, and practices to the broader U.S. population. Responses are subject to recall bias, social desirability bias, and nonresponse bias. Although multiple data quality measures were implemented, Internet surveys may vary in quality and methodology.^29^ Second, survey items were designed for this study and were not previously used to assess cleaning and disinfection. The survey item that assessed cleaning and disinfection safety precautions practiced during the prior month asked about both the respondent and other household members; respondents may not have accurately characterized safety precautions undertaken by household members if they themselves were not involved in cleaning and disinfecting responsibilities. Similarly, assessment of safety precautions practiced during the prior month did not consider the frequency of cleaning and disinfection in the household. Third, data were captured in a cross-sectional manner and do not allow for causal inferences between knowledge, attitudes, and practices. Finally, given the rapid progression of the COVID-19 pandemic and evolving understanding of SARS-CoV-2, responses may not reflect changes in public knowledge, attitudes, and practices over time.

In summary, increasing public knowledge regarding safe cleaning and disinfection, including proper use of and storage of cleaners, disinfectants, and hand sanitizers, will require development and evaluation of effective communication strategies, including those designed to reach specific high-risk demographic groups. Messages such as those found on CDC’s guidance for safe household cleaning and disinfection^9^ or the U.S. Environmental Protection Agency’s “Six Steps for Safe & Effective Disinfectant Use”^30^ may serve as useful tools that can be adapted for these efforts. Additional formative research is needed to develop and test messaging strategies that encourage engagement in safety precautions. Finally, monitoring of changes in knowledge, attitudes, and practices regarding cleaning and disinfection safety over time and across sociocultural settings may inform timeliness and effectiveness of health promotion messages. Whereas cleaning and disinfection of frequently touched surfaces and frequent hand hygiene remain key messages for COVID-19 prevention, messaging should highlight the importance of conducting these activities in a safe and effective manner.

## Supplemental materials

Supplemental materials
