# Supplementary material for: Safe Use and Storage of Cleaners, Disinfectants, and Hand Sanitizers: Knowledge, Attitudes, and Practices among U.S. Adults during the COVID-19 Pandemic, May 2020
Source: Am J Trop Med Hyg. 2020 Dec 29;104(2):496–501. doi: 10.4269/ajtmh.20-1119 (PMC7866329; doi:10.4269/ajtmh.20-1119)
Supplement: Supplementary file 1 [file tpmd201119.SD1.pdf]

Supplementary Materials: Survey Questionnaire

Survey items that collected demographic data or served as data quality filters are not listed. Additionally, seven survey items regarding behaviors specifically intended to prevent SARS-CoV-2 transmission and self-reported health outcomes were not included in the analysis and are not listed.

Survey item 1

Which of the following have you heard is true about using household cleaning products (such as bleach or Lysol)? **[Select as many as apply for 01-09. Randomize 01-09]**

01 For some household cleaning products, gloves should be used during use
02 For some household cleaning products, eye protection should be used during use
03 Bleach should not be mixed with ammonia
04 Bleach should not be mixed with vinegar
05 When making a dilute bleach solution, only room temperature water should be used
06 Hands should be washed with soap and water after using household cleaning products
07 Hand sanitizers should be kept out of reach of children
08 Household cleaning products should be kept out of reach of children
09 Good ventilation (air flow) is needed when using cleaning chemicals
10 None of these **[EXCLUSIVE]**

Survey item 2

In the past month, which of the following behaviors have you, or a household member, engaged in? **[Select as many as apply for 01-10. Randomize 01-10]**

01 Wore gloves when using a household cleaning product (not including sanitizing wipes)
02 Wore eye protection when using a household cleaning product (not including sanitizing wipes)
03 Increased ventilation (air flow) when using a household cleaning product (not including sanitizing wipes)
04 Read the label prior to using a household cleaning product
05 Used hand sanitizer
06 Used sanitizing wipes
07 Used a new type or brand of household cleaning product
08 Prepared homemade cleaning solution (with bleach, ammonia, or vinegar)
09 Labeled a homemade cleaning solution
10 Purchased hand sanitizer or household cleaning product in bulk sizes
11 None of these **[EXCLUSIVE]**

Survey item 3

How much do you agree or disagree with each of the following statements? **[Select one answer for each. Randomize A-F]**

01	02	03	04	05
Strongly disagree	Somewhat disagree	Neither agree nor disagree	Somewhat agree	Strongly agree

A. I know how to clean and disinfect my home safely
B. I am able to clean and disinfect my home safely
C. I know how to store cleaning products in my home safely
D. I am able to buy or locate the household cleaning products I need to clean my home
E. I know where to get information on safe cleaning behaviors
F. People I care about want me to clean my home safely

Survey item 4

How much do you agree or disagree with each of the following statements? **[Select one answer for each. Randomize A-I]**

01	02	03	04	05
Strongly disagree	Somewhat disagree	Neither agree nor disagree	Somewhat agree	Strongly agree

A. For some household cleaning products, gloves should be used during use to prevent injury
B. For some household cleaning products, eye protection should be used during use to prevent injury
C. Washing hands with soap and water after using cleaning household products can prevent injury
D. Keeping hand sanitizer out of reach of children can prevent injury
E. Keeping household cleaning products out of reach of children can prevent injury
F. Misuse of household cleaning products can result in injury
G. Reading the label of household cleaning products prior to use can prevent injury
H. Homemade cleaning solutions should be properly labeled
I. Injuries from misuse of household cleaning products are a common problem
